# Safe and Accurate Sustentaculum Screw Placement in Minimally Invasive Surgery for Calcaneal Fractures: The “Sustentaculum View” Technique

**DOI:** 10.3390/jcm15031228

**Published:** 2026-02-04

**Authors:** Christian Rodemund, Moritz Katzensteiner, Reinhold Ortmaier, Maximilian Vogel, Simon Recheis, Niklas Rodemund, Georg Mattiassich

**Affiliations:** 1 Private Researcher, Alpenblickstrasse 38, 4060 Leonding, Austria; 2 Orthopaedic Department Ordensklinikum Linz, Seilerstätte 4, 4020 Linz, Austria; moritz.katzensteiner@outlook.com (M.K.); r.ortmaier@gmail.com (R.O.); 3 Trauma Center Linz, Garnisonstrasse 10, 4060 Linz, Austria; maximilian.vogel@auva.at (M.V.); simon.recheis@auva.at (S.R.); georg.mattiassich@auva.at (G.M.); 4 Department of Anesthesiology, Perioperative Medicine and Intensive Care Medicine, Paracelsus Medical University, 5020 Salzburg, Austria; niklas@rodemund.net

**Keywords:** sustentaculum screw, calcaneal fracture, minimally invasive surgery

## Abstract

**Background:** The sustentaculum screw plays a crucial role in achieving stable osteosynthesis for intra-articular calcaneal fractures, particularly when using minimally invasive or percutaneous techniques. Accurate placement of the screw within the sustentaculum tali is technically demanding due to the complex anatomy and the limited intraoperative visualization provided by standard fluoroscopic views. **Methods:** Patients were positioned in a standardized lateral decubitus position. Beginning with a standard lateral fluoroscopic view, the C-arm was tilted approximately 25° to align the central beam with the plane of the lower ankle joint. This adjustment enables clear visualization of the borders of the sustentaculum tali and allows precise definition of the target point for guide-wire insertion. To evaluate whether this technique improves screw positioning, two groups were compared: one using the described fluoroscopic view and a control group using conventional imaging alone. **Results:** Screw placement accuracy was significantly higher in the group using the dedicated fluoroscopic view compared with the control group. **Conclusions:** With meticulous preoperative planning, standardized positioning, and the use of a dedicated fluoroscopic setting—referred to as the “sustentaculum view”—accurate and safe screw placement can be achieved with significantly higher accuracy than with conventional imaging alone.

## 1. Introduction

In addition to the surgical approach and reduction technique for calcaneal fractures, osteosynthesis methods vary widely and remain a topic of ongoing discussion. Surgeons use a wide range of fixation methods, including clamps, pins, various types of screws, and numerous plate designs—such as plates specifically developed for the calcaneus as well as plates originally intended for other indications (e.g., radius plates). We observe angle-stable and non-angle-stable plates, solutions for the extensile lateral or sinus tarsi approach, ballons, specific nail systems, and even external fixation techniques [1,2,3,4,5,6,7,8,9,10,11,12].

The choice of the preferred osteosynthesis technique also depends on the surgical approach—plates require larger incisions than screws, increasing the risk of soft tissue complications [13,14], but tend to be more stable [15,16]. However, minimizing complication rates during acute treatment and in long-term outcomes (such as infection, scarring, restricted mobility, and risk of hardware removal) leads to techniques exclusively using screws [2,17,18,19]. An increasing number of scientific studies can be observed in the fields of biomechanics, IT-assisted analysis (finite element analysis) [20], and clinical research. One of the most consistent and fundamental findings is that the clinical outcomes of screw-based treatment are at least comparable to, and in some aspects superior to, those achieved with traditional plate osteosynthesis [21,22].

The sustentaculum tali carries out an important role in the surgical treatment of calcaneal fractures. As a so-called “constant fragment”, it remains stable in its position for nearly all types of calcaneal fractures and serves as an essential reference point for reconstruction. Additionally, the sustentaculum has excellent bone quality, providing a strong mechanical basis for screw fixation. Therefore, it is an essential part of a stabilization concept for calcaneal fractures [23,24,25,26,27,28,29]. However, its small size and anatomical location make reliable intraoperative visualization with conventional methods challenging. The aim of this study was to define optimal screw positioning in relation to anatomy and typical fracture patterns and to introduce a novel imaging technique that standardizes and simplifies accurate screw insertion, as well as to evaluate the clinical effectiveness of this approach.

## 2. Materials and Methods

### 2.1. Anatomy

The sustentaculum is a strong, horizontal bony shelf located on the medial aspect of the calcaneus, supporting the medial portion of the talus. It can be palpated directly beneath the medial malleolus. The inferior surface features a groove for the flexor hallucis longus tendon. The flexor digitorum longus (FDL) is connected to it via its sheaths on the inferomedial side [30], while the tibialis posterior tendon runs cranially. The sustentaculum is securely anchored by several ligaments, notably the spring ligament, which connects it to the navicular bone and supports the talus. Additional stabilization is provided by fibers of the superficial deltoid ligament and the medial talocalcaneal ligament. Bussowitz [26] reported an average height of 12.8 mm (range: 11.3 to 14.4 mm) and a mean width of 20.8 mm, while Li [31] noted a height of 9.5 mm and an average length and width of 23.6 mm.

### 2.2. Own Studies

The complex anatomy of the calcaneus, combined with the limited height of the sustentaculum tali, makes accurate screw placement particularly challenging. In our minimally invasive surgical technique, the procedure is typically performed from the lateral side. Achieving precise intraoperative views of this small structure located on the medial side is therefore difficult.

**Figure 1:**
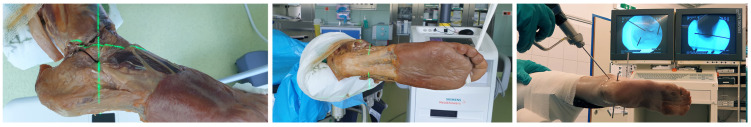
Research on anatomical specimens.

**Figure 2:**
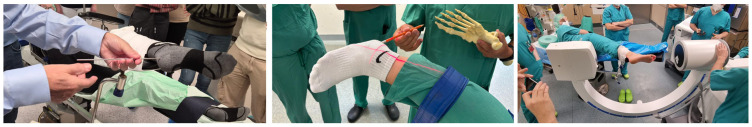
Studies conducted in the operating theatre were performed to define optimal patient positioning and the most effective X-ray views to assist accurate placement of the sustentaculum screw.

To address our requirements, we conducted our own anatomical and technical studies using anatomical specimens (Figure 1), as well as investigations with patients in the operating theater (Figure 2). The results of these studies led to an optimized approach to patient positioning, a standardized X-ray method for all our minimally invasive procedures, and a reliable technique for the placement of sustentaculum screws—which we refer to as the “sustentaculum view” technique.

### 2.3. Clinical Implementation of the “Sustentaculum View” Technique

Unrestricted access to the foot is required to obtain the necessary X-ray views and to allow the use of a distractor with pins extending to the medial side. Based on these requirements, findings from wet-lab studies, and clinical experience, we strongly recommend positioning the patient in a lateral decubitus position. The injured foot is placed posteriorly on a leg holder, with the lateral side facing upward, while the contralateral leg rests on the table in front. Secure fixation is essential, as dorsiflexion of the foot is required intraoperatively to obtain axial views. The foot must be aligned strictly horizontally to ensure consistent positioning and reproducible X-ray views across all procedures.

**Figure 3:**
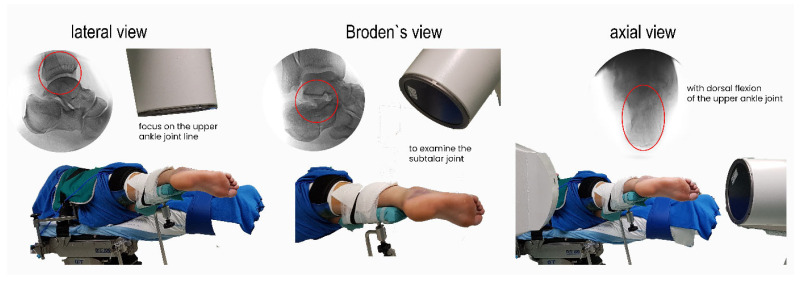
Positioning of the foot and demonstration of the three standard views—lateral, Broden’s, and axial. The lateral view provides essential information on Böhler’s and Gissane’s angles, as well as the length and height of the calcaneus. Precise adjustment of this view is guided by the joint line of the talus (red circle). Broden’s view offers a clear overview of the subtalar joint (red circle). The axial view is required to analyze the frequent angular deviations of calcaneal fragments (red circle). For optimal visualization, additional manual dorsiflexion is required.

The X-ray machine is positioned at the toe end to enable completely free access to the entire foot for the surgeon, especially the heel area. It is aligned exactly in the plane of the foot sole. If an iso-centric machine is available, it allows all three standard views—lateral, Broden’s, and axial—to be obtained from one single fixed position, requiring only movement of the C-arm. The lateral view should focus on the upper ankle joint of the talus, appearing as a clear perfect joint line. Rotating the C-arm by approximately 40 degrees allows the Broden view to be realized for examination of the subtalar joint. Positioning the C-arm horizontally while dorsiflexing the foot reveals the axis of the calcaneus, along with a good view of the sustentaculum from the plantar side (Figure 3).

**Figure 4:**
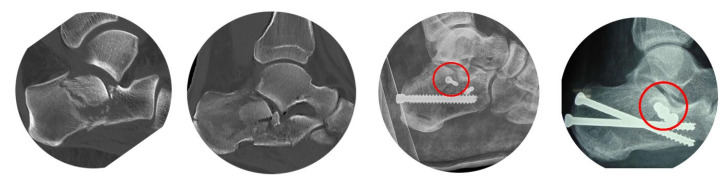
In most of the fractures, we observe a zone of destruction in the center of the calcaneal body. The insertion of screws in this area is not recommended: screws will be either at the edge of a fragment or within a fracture gap.

Selecting an entry point for the sustentaculum screw below the tip of the lateral malleolus is a common cause of insufficient positioning, as illustrated in Figure 4. When analyzing “classical” calcaneal fracture types after falls from height, a primary fracture line is consistently observed in the central region of the calcaneal body. Additionally, we see the Tongue-type or the depression-type fragment, defined through the “secondary fracture line”, in this area. These fragments are more or less depressed with bony defects, cancellous compaction, and frequently some more little fragments. If a screw is inserted laterally in this region, there is a risk of it falling into a fracture gap or contacting the desired fragment only at its edge, potentially leading to tilting or additional fracturing of the fragment.

In order to determine the optimal placement of the screws, a thorough, individual fracture analysis is absolutely essential for each individual case. A CT scan, and particularly a 3D reconstruction, is indispensable.

**Figure 5:**
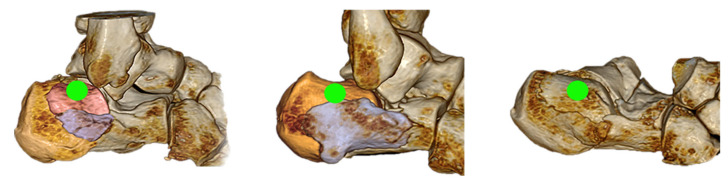
Entry points of the screws, highlighted in green, in the 3D reconstructions. Note that the reduction maneuver results in a fragment shift, leading to a more dorsal and cranial relocation of the screw entry points.

The insertion point through the center of the reduced posterior lateral joint fragment is typically located one to two centimeters horizontally behind the tip of the lateral ankle and should be in the center of the fragment (Figure 5).

**Figure 6:**
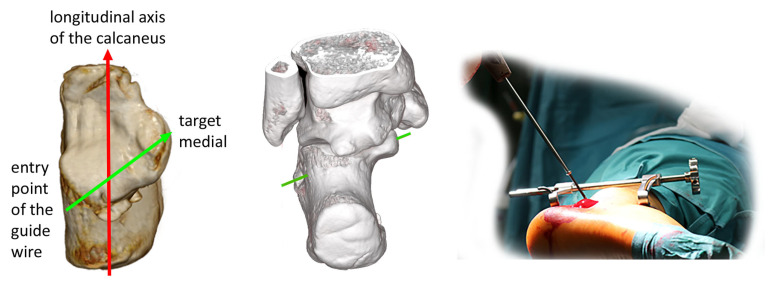
With a starting point behind the lateral ankle, you must angle the guide wire about 30 degrees downward to target the sustentaculum. The green line shows the direction of the guide wire.

These preoperative assessments enable precise identification of the optimal entry point during surgery using an accurate lateral X-ray view. After a small incision, a guide wire is placed on the lateral surface of the calcaneus. It is important to note that the entry point is clearly posterior to the ankle joint, whereas the center of the sustentaculum lies directly beneath the medial malleolus and is therefore considerably more anterior. As a result, the guide wire must be angled approximately 30 degrees downward relative to the transverse axis of the calcaneus (Figure 6).

**Figure 7:**
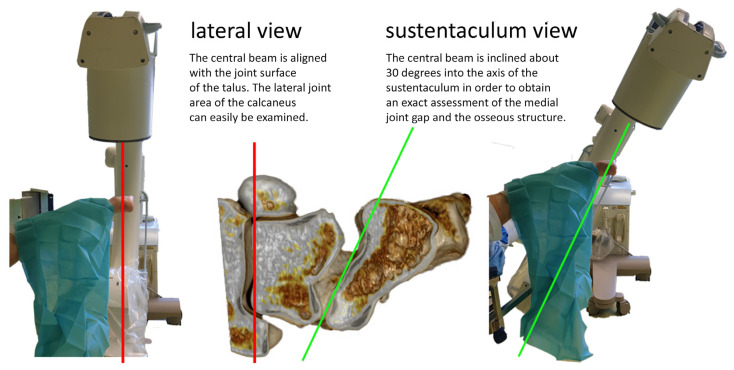
Positioning of the C-arm for the “lateral view” and for the “sustentaculum view”. Red line: plane of the upper ankle joint; green line: plane of the subtalar joint.

At this point, the lateral view is still utilized and is referenced on the joint plan of the talus. The lateral upper margin of the calcaneus will appear slightly distal compared to the medial upper margin. However, this view does not clearly define the shape of the sustentaculum or visualize the posterior subtalar joint gap. It is important to note that the plane of the upper ankle joint and the plane of the posterior subtalar joint are not parallel, forming an angle of approximately 20 to 30 degrees. To achieve an optimal view through the joint, the C-arm must be tilted by this angle (Figure 7).

**Figure 8:**
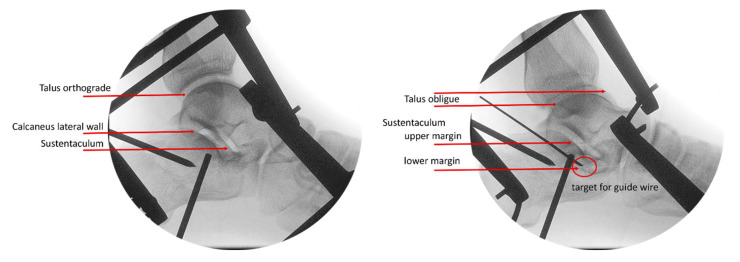
The “sustentaculum view” (right image) shows the outline of the sustentaculum, allowing the target for the guide wire to be defined.

This adjustment provides excellent visualization of the upper and lower borders of the sustentaculum, allowing for accurate target identification and guide-wire insertion (Figure 8). With this setting, there is no risk of inadvertently entering the joint or placing the guide wire below the sustentaculum.

The guide wire’s depth is not a concern because perforation of the skin on the medial side is acceptable, as there are no vulnerable structures in this area. The next step involves positioning the C-arm horizontally, dorsiflexing the upper ankle joint for an axial view, and visualizing the medial part of the sustentaculum. A second pin allows one to measure the screw length. The guide wire can then be transferred over the medial skin level and can be held with a clamp. Final checks should be performed in the lateral, Broden’s, and axial views before finally inserting the screw.

### 2.4. Case Example

**Figure 9:**
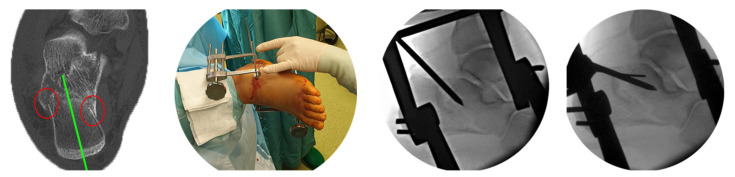
A depression-type fracture with some varus (green line) and shortening (red circles). After distraction we performed the reduction through a dorsi-lateral incision. In the lateral view we defined the entry point for the guide wire of the sustentaculum screw.

Fracture analysis shows a simple depression-type fracture. Because of varus deviation and shortening, we inserted pins into the anterior process of the talus and the tuber calcanei to first correct the axis manually and then lengthen with a distractor. Reduction of the central fragments was performed with a pin through a dorsi-lateral step incision The next step was placement of the guide wire on the lateral wall of the calcaneus, centered within the fragment, under lateral view control (Figure 9).

Then we changed to the “sustentaculum view” to determine the borders of the sustentaculum and to define our target. With this clear visualization it was easy to place the guide wire without the risk of being inside the subtalar joint or below the lower border of the sustentaculum.

**Figure 10:**
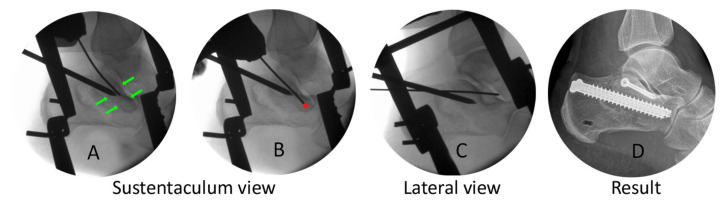
After a change to the sustentaculum view (**A**), we can define the borders of the sustentaculum (green arrows), then determine our target point and drive forward the guide wire ((**B**), red dot). Control in lateral view (**C**). The right picture (**D**) shows the overall result of the surgery.

**Figure 11:**
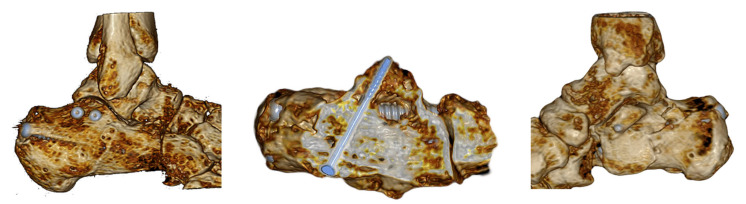
Examples of sustentaculum screws.

Placement was controlled in Broden’s view, then we measured the screw length in axial view, and we finished by inserting the 4.00 mm lag screw (Figure 10). Additional case examples are shown in Figure 11 and in the Supplementary Material, which also includes intraoperative video sequences.

## 3. Results

### 3.1. Calcaneal Fractures and the Sustentaculum

**Figure 12:**
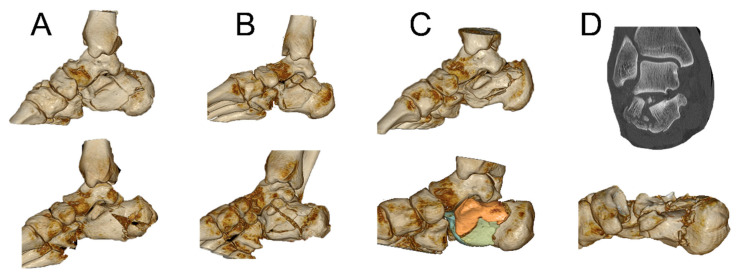
We observe four different situations of the sustentaculum during “classical” calcaneal fractures (defined as fractures after a fall from height, no direct trauma, and no sprain injuries). (**A**) No fracture of the sustentaculum. (**B**) Fracture line through the sustentaculum in an oblique frontal plane—complies with the “primary fracture line” according to the classification of Essex-Lopresti. (**C**) Horizontal fracture below the sustentaculum forming an “uninjured” sustentaculum fragment. (**D**) In severe cases we can find tilting of the sustentaculum; the ligamentous medial fixation remains stable.

Isolated fractures of the sustentaculum are rare and typically require a medial approach and are not the focus of this paper. To verify the suitability of the sustentaculum as a stable structure for our screws, we analyzed its condition in a case series of 380 calcaneal fractures from 2007 to 2020, identifying distinct groups: (A) no involvement of the sustentaculum, usually with a dorsal fracture line, (B) typical frontal fractures without significant displacement, usually central, (C) horizontal fractures in the medial wall near the sustentaculum base, and (D) tilting of the sustentaculum in severe comminuted fractures without a fracture through it (Figure 12).

We never observed a complete dislocation; the sustentaculum remained fixed due to strong ligamentous attachments. Fractures involving the sustentaculum indicate a more severe injury, as demonstrated by our study outcomes: AOFAS scores were 92.00 for cases without fracture, 92.81 for horizontal fractures, 89.54 for fractures through the fragment, and 87.5 for tilting of the sustentaculum. Since the sustentaculum remains stable at its medial fixation, reduction is typically unnecessary and only required in cases of tilting of the fragment. Even the fractures through the sustentaculum did not show any greater gaps and thus a good fixation of the screws can be expected.

### 3.2. Results of Screw Placement with and Without the “Sustentaculum View” Technique

We analyzed the positioning of the sustentaculum screws based on operated calcaneus fractures from 2015 to 2020 at the Trauma Center Linz, Austria. A total of 168 cases were treated with minimally invasive surgery, 125 of which were stabilized using sustentaculum screws (Table 1). Positioning was defined as “perfect” when the screw was located entirely within the sustentaculum tali; rated “tolerable” if the screw lay within 5 mm inferior, anterior, or posterior to the ideal target; and rated “insufficient” when it interfered with the subtalar joint, was more than 5 mm outside the intended goal, or when the lateral entry point was incorrect (see Figure 4). Screw position could be precisely assessed using postoperative CT in 94 fractures; the remaining cases were evaluated by conventional radiographs (lateral, Broden, and axial views). The distribution of ratings (perfect/tolerable/insufficient) did not differ significantly between CT- and X-ray-assessed cases (χ^2^ test, *p* > 0.05), suggesting that imaging modality did not materially influence categorization.

We defined two groups. In the first group, the sustentaculum view technique was used (group 1: 82 cases; 63 perfect, 15 tolerable, and 4 insufficient). The second group consisted of cases in which the technique was not used (group 2: 43 cases; 19 perfect, 16 tolerable, and 8 insufficient). Cases and outcomes were stratified according to the Sanders and Essex-Lopresti classifications.

To compare the two groups with respect to average fracture severity, a point-based scoring system was applied according to the increasing severity of the fracture groups.

Sanders’s type II fractures were assigned one point, type III fractures two points, and type IV fractures three points. The total score was then divided by the number of cases, resulting in an average severity score. With a value of 1.58 to 1.53, it can be said that the two cohorts are comparable. We refer to this method as the Sanders Severity Score.

To compare the groups with respect to the quality of positioning of the screws, a Chi-square test was conducted and the assumptions for the test were checked. It revealed a significant difference between the two cohorts (χ^2^ = 14.19, df = 2, *p* < 0.001). Notably, group 1 showed a higher proportion of “perfect” ratings, whereas group 2 tended to have relatively more “tolerable” and “insufficient” ratings. These findings indicate that the use of the sustentaculum view technique was associated with a significantly higher proportion of optimal screw placements and fewer “insufficient” results.

Outcome distributions also differed significantly among the Sanders fracture groups (χ^2^ = 19.38, df = 10, *p* = 0.035). Sanders type II fractures (2A–2C) showed the highest proportion of perfect outcomes, whereas type III (3AB/3AC) and particularly type IV fractures demonstrated higher rates of acceptable and poor results. This finding was confirmed in a secondary analysis comparing perfect versus non-perfect outcomes (χ^2^ = 14.56, df = 5, *p* = 0.012). We can assume that the severity of the fracture has an influence on the positioning of the screws.

## 4. Discussion

In 1998, Patrick P. Lin [32] presented a laboratory study on various screw configurations combined with a non-angle-stable lateral plate, demonstrating enhanced construct strength when screws were incorporated within the plate. Pang Qing Jiang [33] found that sustentaculum screws provide additional stability for calcaneal fracture fixation in a finite element analysis using a non-locking lateral plate. In a 2015 prospective study, Qiang Min-Fei [28] categorized 119 patients into three groups based on screw placement accuracy. He reported significant height loss in the non-fixation group (Group C) but no differences in AOFAS scores. In 2019, he developed a 3D finite element model [34] showing that precise positioning of screws within or near the sustentaculum does not affect stability in combination with a locking lateral plate, a finding echoed by Li [31] in a clinical study. Rammelt [35] utilized one or two screws in a percutaneous technique described in 2010, engaging the medial cortex to ensure better stability and maintain the anatomical position of the lateral fragment. Our own biomechanical study [36] primarily focused on different combinations of positioning axial static screws and sustentaculum screws. But it seems obvious that the stability of the fixation medially with factors such as bone quality, length of the fixation, penetration of the cortical bone, and compression of the fragments must be of importance for the angular stability of the screw in the lateral region.

The technique for correct positioning is a topic of ongoing discussion. Especially when using plates, visualizing the sustentaculum and achieving the correct angle through the plate holes can be difficult, particularly with locking plates. To enhance success rates, Gras [37] introduced a navigated procedure in 2010. In 2013 Phinit Phisitkul [38] presented a detailed cadaveric study for the optimized placement of screws. Benedict Swartman [39] used a 2-dimensional projection-based software application which detects Kirschner wires and visualizes their intended direction as a colored trajectory. With anatomical studies, 3D simulations, and different fluoroscopic positionings, Liao [40], Sun [41], and Song G [20] tried to find a way for better placement techniques. The main advantage of the “sustentaculum view” technique is that no additional surgical or technical effort is required, and it is solely based on anatomical knowledge and fracture analyses using simple, reproducible, defined steps. Recent technical developments that are becoming standard practice in many specialized centers include the ability to generate intraoperative computed tomography images with minimal additional effort, thereby allowing verification of fracture reduction and osteosynthesis material placement. This will significantly increase the demand for optimal care—and that is good for our patients’ needs.

A limitation of the study is the question of whether the results of group 1 are influenced by the surgeon’s greater experience in treating calcaneal fractures.

## 5. Conclusions

Sustentaculum screws play an important role in stabilizing calcaneal fractures after surgical reduction. With a comprehensive understanding of the calcaneus and subtalar joint anatomy, they can be placed easily and safely. Successful results depend on thorough preoperative fracture analysis, standardized patient positioning, and a special X-ray imaging technique. We refer to this technique of screw placement as the “sustentaculum view.”

## Figures and Tables

**Table 1:** The table shows the two groups with and without using the technique. Cases and results are broken down according to the Sanders and Essex-Lopresti classification. In addition, the average improvement in the Boehler angle is shown for each result group.

Sustentaculum View Technique Used				Results						
		Cases	Percentage of Cases	Sanders Severity Score, Points	Perfect Positioning and Percentage		Tolerable Positioning and Percentage		Insufficient Positioning and Percentage	
	All cases	82			63	76.83	15	18.29	4	4.88
Sanders Classification	2A	32	39.02	32	30	93.75	2	6.25	0	0.00
	2B	10	12.20	10	9	90.00	1	10.00	0	0.00
	2C	1	1.22	1	1	100.00	0	0.00	0	0.00
	3AB	17	20.73	34	10	58.82	5	29.41	2	11.76
	3AC	13	15.85	26	10	76.92	3	23.08	0	0.00
	3BC	0	0.00	0	0	0.00	0	0.00	0	0.00
	4	9	10.98	27	3	33.33	4	44.44	2	22.22
Essex-Lopresti	Tongue-type	29	35.37		18	62.07	8	27.59	3	10.34
	Depression-type	51	62.20		43	84.31	7	13.73	1	1.96
	Atypical	2	2.44		2	100.00	0	0.00	0	0.00
Sanders Severity Score	Average/Score	1.58	total amount	130						
Böhler	Boehler angle preoperative					9.8		3		−16.5
	Boehler angle postoperative					26.68		23.4		8.5
	Improvement of angle					17.84		21.4		25
**Sustentaculum View Technique not Used**				**Results**						
		**Cases**	**Percentage** **of Cases**	**Sanders Severity** **Score** **, Points**	**Perfect Positioning** **and Percentage**		**Tolerable Positioning** **and Percentage**		**Insufficient Positioning** **and Percentage**	
	All Cases	43			19	44.19	16	37.21	8	18.60
Sanders Classification	2A	17	39.53	17	8	47.06	7	41.18	2	11.76
	2B	3	6.98	3	2	66.67	1	33.33	0	0.00
	2C	1	2.33	1	0	0.00	0	0.00	1	100.00
	3AB	20	46.51	40	9	45.00	7	35.00	4	20.00
	3AC	1	2.33	2	0	0.00	0	0.00	1	100.00
	3BC	0	0.00	0	0	0.00	0	0.00	0	0.00
	4	1	2.33	3	0	0.00	1	100.00	0	0.00
Essex-Lopresti	Tongue-type	16	37.21		7	43.75	6	37.50	3	42.86
	Depression-type	24	55.81		10	41.67	10	41.67	4	40.00
	Atypical	3	6.98		2	66.67	0	0.00	1	50.00
Sanders Severity Score	Average/Score	1.53	total amount	66						
Bohler	Boehler angle preoperative					12.78		5.84		5.52
	Boehler angle postoperative					27.26		21.84		15.89
	Improvement of angle					14.48		16		10.36

## Data Availability

The raw data supporting the conclusions of this article will be made available by the authors on request.

## Supplementary Materials

The following supporting information can be downloaded at: https://drive.google.com/file/d/11Cj3qF0r_IVB9A8eKKUxm_li1x_QSdG2/view?usp=drive_link accessed on 30 December 2019. Website: www.calcaneal-fracture.com.

## Abbreviations

The following abbreviations are used in this manuscript:

AOFAS	American Orthopaedic Foot and Ankle Society Ankle-Hindfoot Scale
